# Surgical resection and radiofrequency ablation initiate cancer in cytokeratin-19^+^- liver cells deficient for p53 and Rb

**DOI:** 10.18632/oncotarget.9952

**Published:** 2016-06-13

**Authors:** Ramadhan B Matondo, Mathilda JM Toussaint, Klaas M Govaert, Luciel D van Vuuren, Sathidpak Nantasanti, Maarten W Nijkamp, Shusil K Pandit, Peter CJ Tooten, Mirjam H Koster, Kaylee Holleman, Arend Schot, Guoqiang Gu, Bart Spee, Tania Roskams, Inne Borel Rinkes, Baukje Schotanus, Onno Kranenburg, Alain de Bruin

**Affiliations:** ^1^ Department of Pathobiology, Faculty of Veterinary Medicine, Utrecht University, Utrecht, The Netherlands; ^2^ Department of Surgical Oncology, Cancer Centre, UMC Utrecht, Utrecht, The Netherlands; ^3^ Program in Developmental Biology and the Department of Cell and Developmental Biology, Vanderbilt University Medical Center, Nashville, TN, USA; ^4^ Department of Clinical Sciences of Companion Animals, Faculty of Veterinary Medicine, Utrecht University, Utrecht, The Netherlands; ^5^ Translational Cell and Tissue Research, University of Leuven, Leuven, Belgium; ^6^ Department of Pediatrics, Division of Molecular Genetics, University Medical Center Groningen, University of Groningen, Groningen, The Netherlands

**Keywords:** liver, cholangiocytes, inflammation, necrosis, mice

## Abstract

The long term prognosis of liver cancer patients remains unsatisfactory because of cancer recurrence after surgical interventions, particularly in patients with viral infections. Since hepatitis B and C viral proteins lead to inactivation of the tumor suppressors p53 and Retinoblastoma (Rb), we hypothesize that surgery in the context of p53/Rb inactivation initiate *de novo* tumorigenesis.

We, therefore, generated transgenic mice with hepatocyte and cholangiocyte/liver progenitor cell (LPC)-specific deletion of p53 and Rb, by interbreeding conditional *p53/Rb* knockout mice with either *Albumin-cre* or *Cytokeratin-19-cre* transgenic mice.

We show that liver cancer develops at the necrotic injury site after surgical resection or radiofrequency ablation in p53/Rb deficient livers. Cancer initiation occurs as a result of specific migration, expansion and transformation of cytokeratin-19^+^-liver (CK-19^+^) cells. At the injury site migrating CK-19^+^ cells formed small bile ducts and adjacent cells strongly expressed the transforming growth factor β (TGFβ). Isolated cytokeratin-19^+^ cells deficient for p53/Rb were resistant against hypoxia and TGFβ-mediated growth inhibition. CK-19^+^ specific deletion of p53/Rb verified that carcinomas at the injury site originates from cholangiocytes or liver progenitor cells.

These findings suggest that human liver patients with hepatitis B and C viral infection or with mutations for p53 and Rb are at high risk to develop tumors at the surgical intervention site.

## INTRODUCTION

Liver cancer is one of the deadliest forms of cancer with approximately 700,000 deaths per year worldwide [1]. Hepatic resection and radiofrequency ablation (RFA) are the treatment of choice, especially for patients with tumors associated with early and severe cirrhosis respectively [2, 3]. However, long-term prognosis after resection or RFA of liver cancer remains unsatisfactory, because of the high recurrence rate [4, 5]. A retrospective study revealed that the 5-year recurrence rates after surgery (n=138) and RFA (n=236) were 53.7% and 69.5% respectively [6].

Many risk factors have been associated with liver cancer including chronic hepatitis B and C viral infection, and basically all cirrhosis-inducing conditions [7]. These etiological exposures are believed to interfere with the cell cycle machinery through inactivating tumor suppressor pathways [8]. Among the pathways that are often disrupted in concert are those regulated by the tumor suppressors *p53* and *Rb* [9–11]. For example, viral proteins derived from Hepatitis B and C viruses have been shown to inactivate *p53* as well as *Rb* in liver cells [12, 13]. Interestingly, liver specific deletion of the *Rb* gene in mice does not result in spontaneous tumor development [14], whereas deletion of all three pocket proteins (Rb, p107, p130) led to spontaneous liver cancer, indicating compensatory tumor suppressor mechanisms within the pocket protein family [15]. Deletion of *p53* in mice is sufficient to cause spontaneous liver cancer [16, 17]. Intriguingly, liver-specific deletion of both *p53* and *Rb* did not result in spontaneous liver cancer in mice aged to one year. However, in response to diethylnitrosamine (DEN), tumors started to develop at the age of three months [17, 18].

Previous studies in humans demonstrated that events that lead to the inhibition of the p53/Rb pathways occurred at early stages of the disease, indicating that these tumor suppressor pathways might play a critical role in preventing liver cancer initiation [19].

Hepatocytes, cholangiocytes and liver progenitor cells could function as candidates for the cell of origin in liver cancer [20]. However, identifying the cell of origin is currently not feasible in human patients. In addition, the lack of appropriate markers to clearly distinguish the differentiation stages of the different hepatic lineages has hindered the characterization of the cell of origin in human liver cancer patients [21]. In this study, we show that liver-specific inactivation of Rb and p53 in mice leads to the spontaneous formation of liver tumors at the age of 13 months with histological similarities to human liver cancers. Moreover, we show that surgical resection and RFA accelerate tumor genesis in p53/Rb deficient livers as a result of migration, expansion and transformation of Cytokeratin19- (CK19) positive - cells at the injury site.

## RESULTS

### Loss of p53 and Rb in liver results in spontaneous cancer

We used homologous recombination techniques and *Cre-loxP* technology to disrupt *p53* and *Rb* function in the liver by crossing *Alb-cre to p53^f/f^*;Rb*^f/f^* mice (Supplementary Figure S1A and S1B).

**Figure 1:**
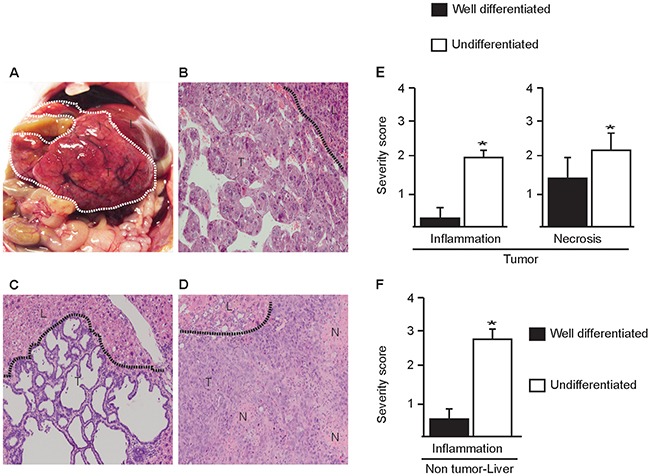
Liver specific loss of p53 and Rb in mice results in spontaneous liver tumors **A.** *Insitu* view of a liver tumor in the abdomen of *Alb-cre^+/−^*; p53*^Δ/Δ^*; Rb*^Δ/^*^Δ^ mouse, **B.** well differentiated HCC, **C.** well differentiated CC, **D.** undifferentiated HCC/CC with inflammation and necrosis(N). Dotted lines indicate borders between tumor **T.** and normal liver (L). Histological images are magnified 100x. Quantification of inflammation and necrosis; **E.** in well differentiated and undifferentiated HCC/CC and **F.** in non-tumor liver tissues adjacent to neoplastic tissues. Data represent average ± s.e.m, (n= 10). *p <0.05, undifferentiated versus well differentiated HCC/CC.

Homozygous deletion of *p53* and *Rb* in the liver led to spontaneous tumor formation in 13-26 month-old mice with an incidence of 63% (n=19) (Figure 1A; Table 1). No liver tumors were observed in age-matched control mice (n=17). Analysis of tumors discovered different phenotypes including well-differentiated hepatocellular carcinoma (HCC, Figure 1B), cholangiocarcinomas (CC, Figure 1C) and undifferentiated hepatocholangiocellular carcinomas HCC/CC (Figure 1D). Importantly undifferentiated carcinomas contained multiple areas with coagulation necrosis (Figure 1E) accompanied by infiltration of inflammatory cells (Figure 1D, Supplementary Figure S1C). The degree of necrosis and inflammation was more severe in undifferentiated HCC/CC compared to differentiated HCC/CC (Figure 1E). Notably the liver surrounding undifferentiated tumors displayed also more infiltration of inflammatory cells compared to well-differentiated tumors (Figure 1F). These findings suggest that loss of epithelial differentiation is a consequence of tissue injury and its associated inflammatory response. Alternatively, since undifferentiated carcinomas exhibited more necrosis this might trigger an enhanced immune response.

**Table 1:** Life span, tumor incidence and tumor differentiation of mice with the indicated genotypes and types of surgical interventions

		Mean life span	Tumor at surgery site	EMT	Tumor not at surgery site	EMT
1	*Alb-cre^−/−^ ;p53^f/f^;Rb^f/f^*; (with surgery)	730	0% (0/17)	n.a.	0% (0/17)	n.a.
2	*Alb-cre^+/+^ ;p53^Δ/Δ^;Rb^Δ/Δ^*(no surgery)	527*	n.a.	n.a.	63% (12/19)	66% (8/12)***
3	*Alb-cre^+/+^;p53^Δ/Δ^;Rb^Δ/Δ^* (PH)	229**	66% (23/35)	100% (23/23)	6% (2/35)	0% (0/2)
4	*Alb-cre^+/+^;p53^Δ/Δ^;Rb^Δ/Δ^* (RFA)	n.a.	100% (16/16)	100% (16/16)	0% (0/16)	n.a.
5	*Ck19-cre^+/+^;p53^Δ/Δ^;Rb^Δ/Δ^* (RFA)	n.a.	42% (10/24)	100% (10/10)	0% (0/24)	n.a.

Group 2 has a significant lower mean life span compared to group 1 (*P<0.05); group 3 has a significant lower mean life span compared to group 2 (**P< 0.001; SPSS log rank test). Epithelial-mesenchymal transition (EMT) in tumors located at surgery site of group 3 is significantly higher compared to tumors of group 2 (***P<0.01; two sided students t-test). PH, partial hepatectomy; RFA, radiofrequency ablation; n.a., not applicable.

### Hepatic resection leads to cancer at the ligation site in *p53*^Δ/Δ^;*Rb*^Δ/Δ^ livers

**Figure 2:**
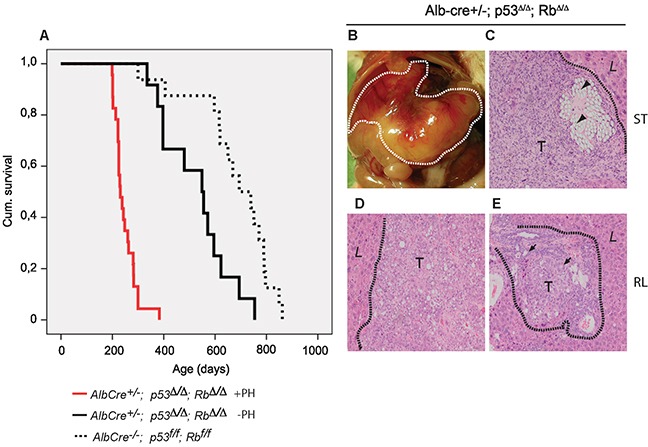
Hepatic resection in *Alb-cre^+/−^*; p53*^Δ/Δ^*; Rb*^Δ/Δ^* mice results in reduced survival and undifferentiated carcinomas at the ligation site **A.** Survival curves of *Alb-cre^+/−^*;p53*^Δ/Δ^*;Rb*^Δ/Δ^* mice developing liver tumors after (+PH; red) or without (−PH; black) partial hepatectomy. Life span of control mice is indicated by dotted line. **B.** View of a liver tumor after PH. **C.** H&E (100x) stained liver from undifferentiated carcinomas within the remaining stump (ST). Suture material (arrowheads) localized in neoplastic lesions. **D.** Well differentiated HCC and **E.** CC with moderately differentiated bile ducts (arrows) in regenerating lobes (RL). Dotted lines separate normal liver (L) and tumor (T).

Survival analysis demonstrated that the mean life span of *Alb-cre*^*+/−*^*;p53*^*Δ/Δ*^*;Rb*^*Δ/Δ*^ mice that developed spontaneous liver tumors (527 days; n=12) was significantly shorter than control *Alb-cre*^*−/−*^*;p53*^*f/f*^*;Rb*^*f/f*^ mice (730 days; n=17; *P*=<0.05) (Figure 2A; Table 1). Although deletion of *p53* and *Rb* is already induced during fetal development [22], tumor formation occurred relatively late in life, indicating that additional events are required to initiate cancer. To investigate whether tumor development could be accelerated in *p53*^*Δ/Δ*^*;Rb*^*Δ/Δ*^ liver, we performed two subsequent partial hepatectomies (PH). Surgical resection resulted in early formation of large liver tumors with an incidence of 66% (Table 1). The mean survival time of Alb-*cre*^*+/−*^*;p53*^*Δ/Δ*^*;Rb*^*Δ/Δ*^ mice that underwent surgery and developed liver tumors was significantly shorter compared to tumor mice without surgery (Figure 2A; Table 1). We expected that liver tumors would be located in the regenerating lobes, because these lobes underwent multiple rounds of cell division, which would likely increase the risk of gaining additional mutations. Surprisingly, in all *p53*^*Δ/Δ*^*;Rb*^*Δ/Δ*^ livers a large tumor was found at the stump of the resected lobes (Figure 2B), whereas in the regenerating lobes hardly any liver tumors were detected. Histological examination revealed that all tumors located at surgical sites were undifferentiated HCC/CC accompanied with multifocal necrosis and infiltration of inflammatory cells (Figure 2C, Table 1). Tumors that developed occasionally in a regenerated lobe away from the injury site were more well-differentiated HCC or CC (Figure 2D, 2E; Table 1). PH on control mice did not cause liver cancer (Supplementary Figure S2).

### RFA initiates cancer at the necrotic injury site of *p53*^Δ/Δ^;*Rb*^Δ/Δ^ livers

**Figure 3:**
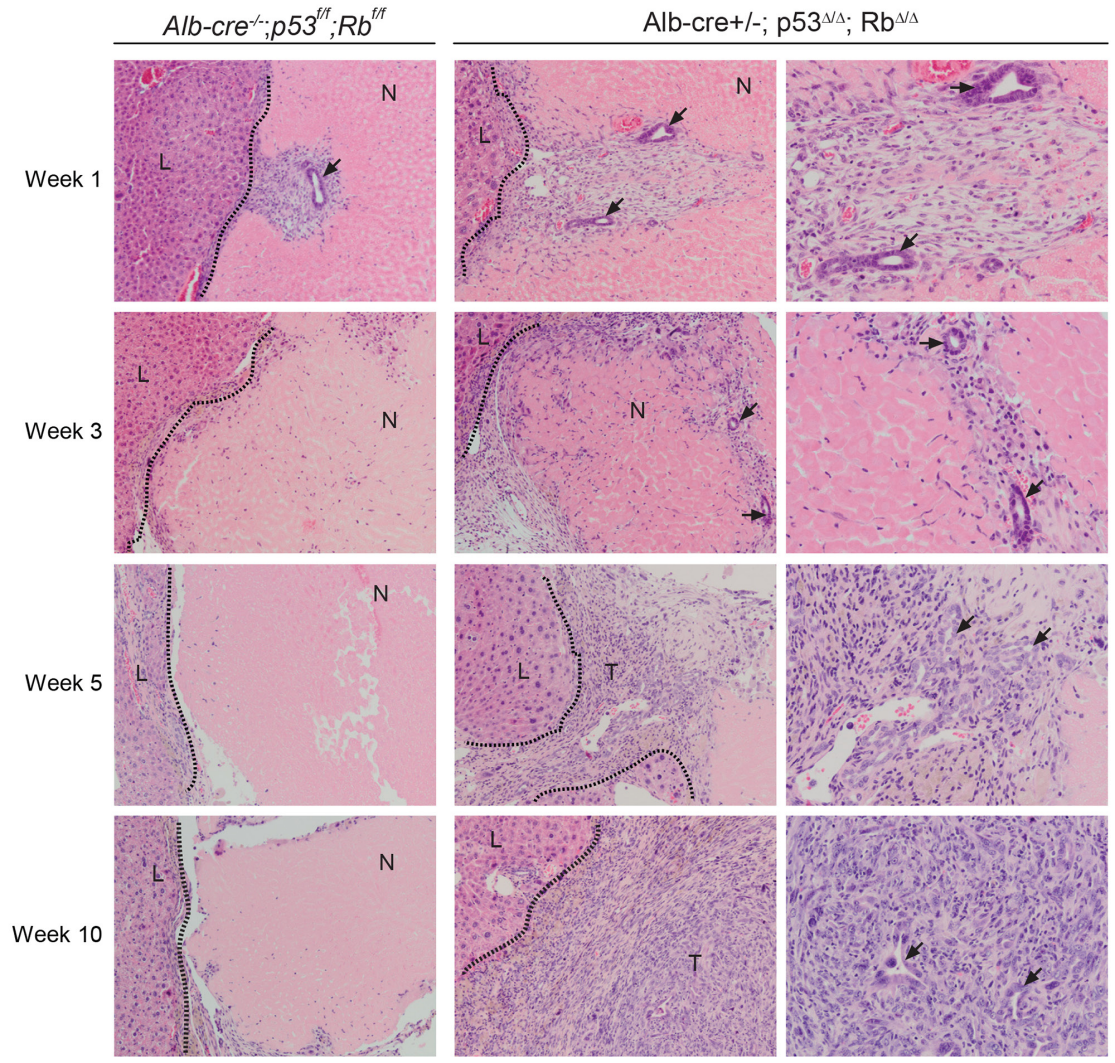
RFA *in Alb-cre^+/−^*; p53*^Δ/Δ^*; Rb*^Δ/Δ^* mice leads to expansion of bile duct cells and formation of liver tumors at the injury site Representative H&E liver sections from necrotic lesions of *Alb-cre^+/−^*; p53*^Δ/Δ^*; Rb*^Δ/Δ^* mice and control *Alb-cre^−/−^*;p53*^f/f^*;Rb*^f/f^* mice at different time points after RFA. Migration of bile ducts (arrows) and appearance of liver tumors (T). Dotted lines mark the border between liver (L) and necrotic RFA lesion (N). Left and middle panel 100x; right panel 200x.

We evaluated whether RFA, a different therapy approach to remove tumors from the liver, had any effects on *p53^Δ/Δ^*;Rb*^Δ/Δ^* and control *p53^f/f^*;Rb*^f/f^* livers. Mice were euthanized weekly for post RFA analysis from 1-10 weeks (Supplementary Figure S3A). Examination of the injured liver lobes revealed that distinct neoplastic foci were detectable within the necrotic regions of *p53^Δ/Δ^*;Rb*^Δ/Δ^* and not in control livers from 5 weeks after RFA (Figure 3, Table 1). Tumors that developed upon RFA treatment were similar to tumor induced by PH and were classified as undifferentiated HCC/CC (Figure 3 and 2B, 2C; Table 1). In *Alb-cre^+/−^*;p53*^Δ/Δ^*, 4/5 mice developed tumors within 6 months, whereas no tumor was observed in *Alb-cre^+/−^*;Rb*^Δ/Δ^* mice within 12 months after RFA treatment. Morphologically, tumors observed in *p53^Δ/Δ^* liver were identical to *p53^Δ/Δ^*;Rb*^Δ/Δ^* tumors (data not shown).

### CK19^+^ cells migrate, expand and transform within necrotic regions of *p53*^Δ/Δ^;*Rb*^Δ/Δ^ livers

**Figure 4:**
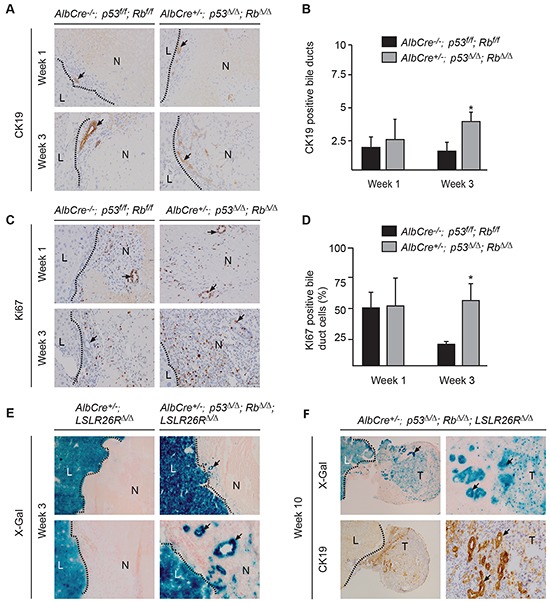
Loss of *p53* and *Rb* leads to enhanced proliferation cholangiocytes within the RFA site **A.** CK19 positive and **B.** quantification of bile ducts. **C.** Ki67-positive cholangiocytes and **D.** quantification of Ki67 positive cholangiocytes. [(A), (C)] Images magnified 200x. Histograms represent average ± s.d. of five fields at 20x, (n= 2-4), *p <0.05 versus control. **E.** x-gal, and expansion of p53/Rb-deficient bile ducts (arrows). Upper panel 100x, lower panel 200x. **F.** X-gal and CK19 staining 10 weeks after RFA. Bile ducts (arrows) within the tumors (T), left panel 40x, right panel 200x. Viable liver (L) and necrotic (N) tissue.

Next we investigated which cell types invaded into the necrotic region before distinct neoplastic foci were recognizable. One week after RFA, we observed in *p53^Δ/Δ^*;Rb*^Δ/Δ^* and in control livers that multiple well differentiated bile ducts were present within the necrotic region (Figure 3). These bile ducts were surrounded by myofibroblasts, inflammatory cells and endothelial cells and localized adjacent to the border between dead and viable liver tissue. Intriguingly, at 3 weeks these bile ducts and the surrounding cells had migrated and expanded deeper into the necrotic liver regions in *p53^Δ/Δ^*;Rb*^Δ/Δ^* and not in control liver. Notably, viable well-differentiated hepatocytes had never been detected within necrotic regions (Supplementary Figure S3B). Presence and expansion of bile ducts within the necrotic region was confirmed by immunostaining for CK19, a marker for cholangiocytes and liver progenitor cells (LPC) (Figure 4A). Number of bile ducts (Figure 4B) and proliferation index of cholangiocytes (Figure 4C & 4D) in *p53^Δ/Δ^*;Rb*^Δ/Δ^* liver were increased 3 weeks after RFA. These findings suggest that liver-specific loss of p53 and Rb results in enhanced migration and proliferation of bile ducts into the area of tissue injury. Remarkably, 5-6 weeks post RFA and at later time points, bile duct structures were still present within the neoplastic foci surrounded by inflammatory cells, but were smaller in size (Figure 3).

The *Alb-cre* transgene is known to specifically express Cre in hepatocytes. To verify that homologous recombination of conditional alleles had also occurred in cholangiocytes/LPC and not in hepatocytes only, we crossed *Alb-cre^+/−^*;p53*^Δ/Δ^*;Rb*^Δ/Δ^* mice with mice carrying the *LacZ-Cre*-reporter (*LSLR26R^f/f^*) [23]. As expected LacZ expression was detected in viable hepatocytes of *Alb-cre*^+/™^; *LSLR26R^Δ/Δ^* mice (Figure 4E). Importantly, bile ducts in non-injured livers (Supplementary Figure S1B) and bile ducts invading early into the necrotic regions of *Alb-cre^+/−^*;p53*^Δ/Δ^*;Rb*^Δ/Δ^*;LSLR26R*^Δ/Δ^* mice showed strong LacZ expression (Figure 4E), indicating that *p53* and *Rb* has not only been deleted in hepatocytes but also in cholangiocytes/LPC. LacZ expression was also observed in liver tumors 10 weeks after RFA, and we found that undifferentiated carcinomas cells and cholangiocytes/LPC expressed LacZ, supporting that undifferentiated neoplastic cells originate from epithelial liver cells (Figure 4F). Deletion of *p53* and *Rb* in liver tumors was confirmed by PCR analysis (Supplementary Figure S1A).

### *p53*^Δ/Δ^;*Rb*^Δ/Δ^ CK19^+^ cells are resistant to TGFβ and hypoxia mediated growth inhibition

**Figure 5:**
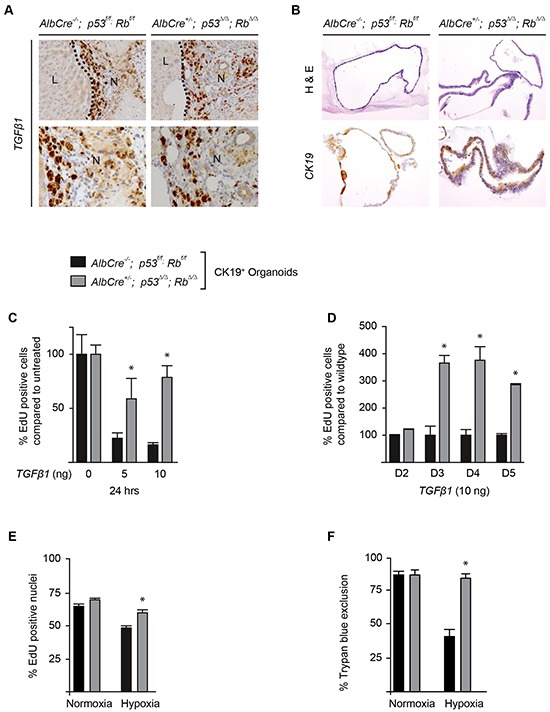
*p53* and *Rb* deficient bile ducts are resistant to proliferation inhibitory signals induced by TGFβ1 and hypoxia **A.** *TGFβ1* expression in liver **(L)** and necrotic tissue (N), 3 weeks after RFA. Magnifications: 200x (top row) and 400x (bottom row). **B.** H&E (top row) and CK19 (bottom row) on bile duct organoids of indicated genotypes, all images at 200x magnification. **C.** Proliferation of organoids from wild type and p53/ Rb deficient livers at an increasing dosage of TGFβ1. **D.** Comparison of proliferation rate of wild type versus p53/Rb knockout organoids cultured for 5 days with and without TGFβ1 treatment. **E.** Proliferation rate of control versus p53/ Rb deficient organoids under normoxia and 2.5% hypoxia for 24 hours. **F.** Survival score of organoids under normoxic and hypoxic conditions shown by trypan blue exclusion in control versus p53/ Rb deficient organoids. Histograms represent mean and standard deviations, *p < 0.05, wild type versus p53/Rb deficient cells.

Since the proliferation rate of wild type cholangiocytes within the RFA zone decreased from 1 to 3 weeks (Figure 4C, 4D) while *p53/Rb* deficient cholangiocytes continue to proliferate at high rate, we hypothesize that *p53^Δ/Δ^*; Rb*^Δ/Δ^* cholangiocytes might be less responsive towards inhibitory proliferation signals, such as the transforming growth factor beta (TGFβ). TGFβ is a secreted protein and a known inhibitor of cellular proliferation [24, 25]. We found that TGFβ is highly expressed by cells surrounding the migrating cholangiocytes at the RFA site 3 weeks after surgery (Figure 5A). To test our hypothesis, we isolated and cultured cholangiocytes/LPC from *p53^Δ/Δ^*;Rb*^Δ/Δ^* and wild type livers using organoid technology (Figure 5B) [26]. Administration of TGFβ to the culture medium of wild type organoids strongly reduced the DNA replication rate, quantified by EdU incorporation (Figure 5C). In contrast, TGFβ addition to *p53^Δ/Δ^*;Rb*^Δ/Δ^* organoids resulted in less inhibition of DNA replication (Figure 5C). Time course analysis with daily measurements of DNA replication further supported that *p53^Δ/Δ^*;Rb*^Δ/Δ^* organoids were less sensitive to inhibitory effects and displayed a higher proliferation rate in response to TGFβ (Figure 5D). Similar responses were observed when CK-19^+^ organoids were cultured under hypoxic conditions. Hypoxia decreased the proliferation rate and viability of wild type liver organoids, whereas *p53^Δ/Δ^*;Rb*^Δ/Δ^* organoids show no reduction in proliferation and viability (Figure 5E, 5F). To investigate whether oxidative stress contributes to the positive selection of CK19^+^ cells after RFA [27], we exposed organoids to hydrogen peroxide or we removed the anti-oxidant N-Acetyl-Cysteine (NAC) from the organoid medium. In contrast to hypoxia and TGFβ, wild type and *p53^Δ/Δ^*;Rb*^Δ/Δ^* organoids did not differ significantly in response to oxidative stress (Supplementary Figure S9A-S9D). Next, we evaluated the angiogenesis response towards the injury. However the number of blood vessels at the transition zone two weeks after RFA did not differ between control and p53/Rb deficient livers (Supplementary Figure S10A-S10B). Furthermore, the analysis of the polyploidy status of isolated wild type and p53/Rb deficient CK19^+^ cells from livers, revealed no difference in polyploidy between the two genetic groups (Supplementary Figure S11).

### Tissue destruction leads to epithelial-mesenchymal transition (EMT) of *p53*^Δ/Δ^;*Rb*^Δ/Δ^ neoplastic liver cells

**Figure 6:**
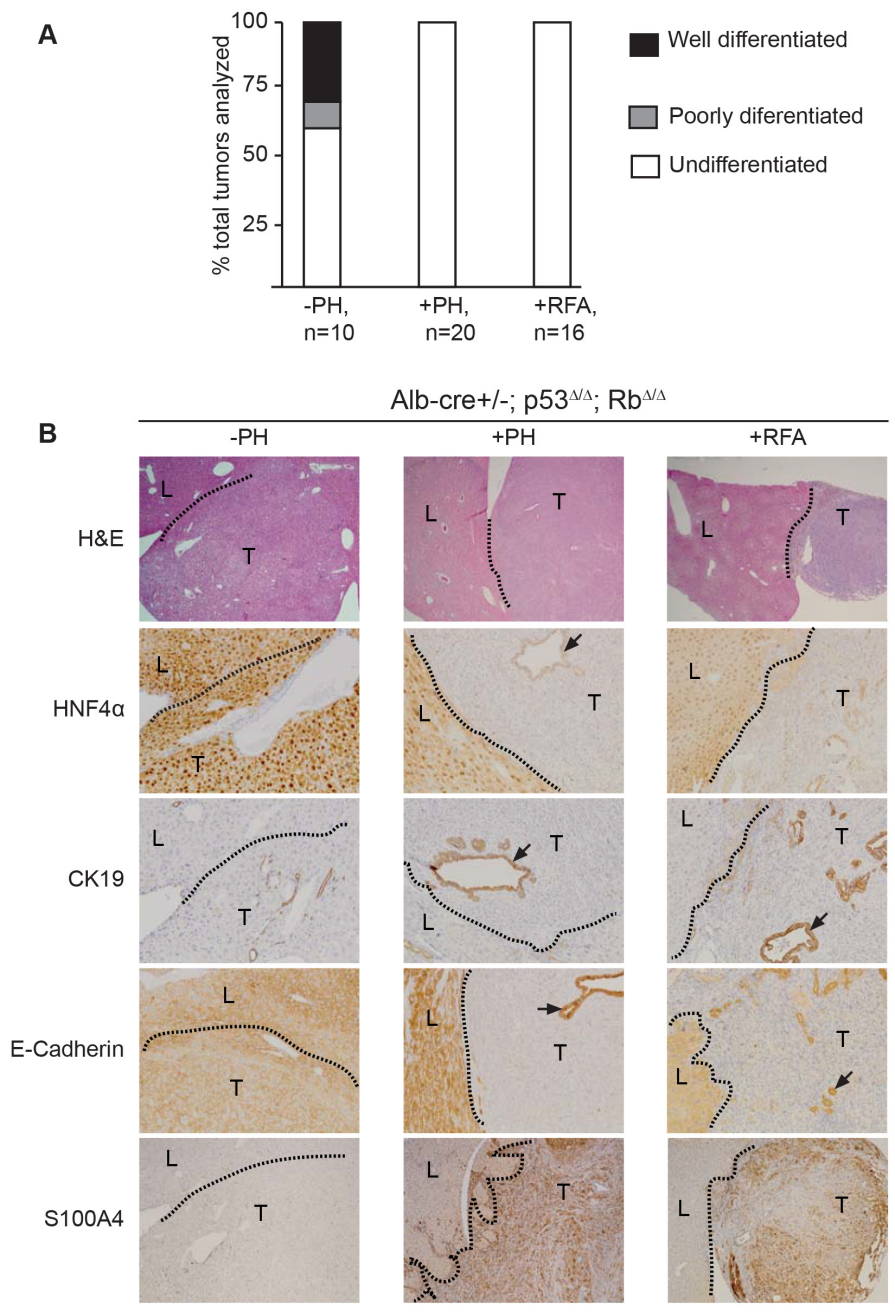
Surgical resection or RFA in *Alb-cre^+/−^*; p53*^Δ/Δ^*; Rb*^Δ/Δ^* mice lead to formation of undifferentiated carcinomas that underwent epithelial-mesenchymal transition **A.** Classification of spontaneous liver tumors (−PH), tumor associated with partial hepatectomy (+PH) or radiofrequency ablation (+RFA), and number of tumors analyzed (n). **B.** Images of H&E (20x), hepatocyte nuclear factor 4α (HNF4α, 100x), CK19 (100x), E-cadherin (100x), and S100A4 (40x) and magnifications for the respective images are shown in brackets.

Analysis of all spontaneous tumors arising in *p53*^*Δ/Δ*^*;Rb*^*Δ/Δ*^ livers revealed that approximately 30% of tumors were well differentiated HCC or CC, while the remaining tumors were either poorly differentiated or undifferentiated (Figure 6A). Reduced differentiation of spontaneous tumors was associated with the presence of prominent necrotic foci and strong invasion of inflammatory cells (Figure 1E). Consistent with these observations, 100% of the liver tumors that developed after PH or RFA were undifferentiated HCC/CC (Figure 6A; Table 1). These findings prompt us to hypothesize that tissue destruction induced EMT of neoplastic liver cells. Selected markers were used to investigate the differentiation degree of the liver tumors in more detail. Hepatocyte nuclear factor 4 alpha (HNF4α), a nuclear marker for mature hepatocytes [28], was strongly expressed in normal hepatocytes and well differentiated HCC, whereas undifferentiated carcinomas showed almost no expression (Figure 6B). We observed that bile ducts located within undifferentiated carcinoma showed weak expression of HNF4α, which is in line with previous studies describing HNF4α expression in activated bile ducts (Figure 6B) [28]. CK19 staining displayed strong expression in normal bile ducts, in well differentiated CC, and in large and small ductular structures within undifferentiated carcinomas (Figure 4F, 6B, data not shown). Undifferentiated carcinoma cells showed infrequent low expression of CK19 (Figure 4F, 6B). Next, we investigated the expression of E-cadherin, a general marker for epithelial cells. Strong membranous E-cadherin expression was observed in normal hepatocytes and cholangiocytes, in well differentiated HCC and CC, and in ducts of undifferentiated carcinomas. Consistent with the expression pattern of the other epithelial marker HNF4α and CK19, undifferentiated carcinoma cells lacked E-cadherin expression (Figure 6B and Supplementary Figure S4). To determine whether these carcinomas cells had gained a mesenchymal phenotype, we performed S100A4 staining. Undifferentiated carcinomas cells show strong S100A4 expression, whereas bile ducts and hepatocytes lacked S100A4 expression (Figure 6B and Supplementary Figure S4). We also observed co-localization of CK19 and S100A4 as well as CK19 and vimentin in early tumor lesions (Supplementary Figure S5A, S5B). Other regions of advanced tumor lesions and normal liver tissues did not show co-expression of epithelial and mesenchymal markers (Supplementary Figure S6A, S6B).

Finally, Ki67 staining demonstrated enhanced Ki67 expression in undifferentiated carcinomas (Supplementary Figure S6C). Together these data suggest that tissue destruction by surgical resection or RFA leads to epithelial-mesenchymal transition and enhanced proliferation of *p53^Δ/Δ^*; Rb*^Δ/Δ^* neoplastic cholangiocytes/LPC.

### Anti-inflammatory drugs could not prevent tumor development at the RFA injury site

Because the initiation of liver tumors at the injury site is associated with infiltration of inflammatory cells (Figure 3), we investigated whether inflammation contributes to tumor development. One group of *Alb-cre^+/−^*;p53*^Δ/Δ^*;Rb*^Δ/Δ^* mice received Sulindac in drinking water and control group received plain water. A second cohort group received Dexamethasone or saline a day before RFA and continued treatment for 9 weeks. The Sulindac treated group (n=6) did not differ from the control group. Dexamethasone treated mice revealed reduced inflammation and bile ducts migration into necrotic region, three weeks after RFA (Supplementary Figure S7). Surprisingly only one mouse of the dexamethasone treated group did not develop tumors at RFA site, 75% (6/8) developed smaller tumors compared to control and one mouse developed a tumor larger than the control mice. As expected, all saline treated mice developed tumors at RFA site. Treatment with high dose of Dexamethasone (4mg/kg) alone did not induce any pathological change in the liver (data not shown). These findings suggest that reducing the inflammatory response at the injury site does not significantly contribute to the development of liver tumors after RFA.

### CK19-specific deletion of p53 and Rb leads to liver cancer after RFA

**Figure 7:**
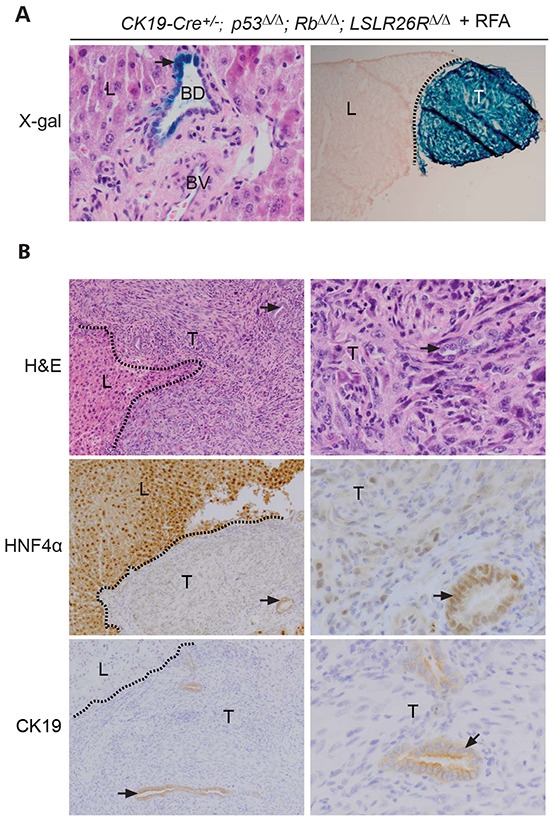
Cholangiocyte specific deletion of *p53* and *Rb* in mice leads to undifferentiated carcinomas at the RFA injury site **A.** Left panel: in situ x-gal positive bile ducts cells (BD, arrows) at 400x magnification in normal liver (L): portal fibroblasts, blood vessels (BV) and hepatocytes were not stained. Right panel: x-gal positive liver tumor at RFA injury site (20x). **B.** HNF4α and CK19 in liver and tumors. The presence of bile ducts (arrows) within the tumors is indicated. Dotted lines separates normal liver tissue (L) and tumors (T). Left panel, 100x, right panel 400x.

Given the migration and expansion of cholangiocytes into early necrotic lesions of p*53^Δ/Δ^*;Rb*^Δ/Δ^* livers (Figure 3), we examined whether the bile duct cell/LPC could represent the cell-of-origin in this injury-induced mouse model of liver cancer. To delete *p53* and *Rb* specifically in cholangiocytes/LPC, we bred mice transgenic for *Cre* under the control of the tamoxifen-inducible *CK19* promoter (*CK19-creERT*) [29] with *p53^f/f^*;Rb*^f/f^* mice. To monitor *Cre*-specific expression, mice were also crossed with the *R26LSL-lacZ* reporter mice. 5-bromo-4-4chloro-3-indolyl-D-galacto-pyrmoside (X-gal) staining on liver sections from *CK19-cre^+/−^*;p53*^Δ/Δ^*;Rb*^Δ/Δ^*;LSLR26R*^Δ/Δ^* mice injected with tamoxifen demonstrated LacZ expression in cholangiocytes, not in hepatocytes or stromal cells (Figure 7A). To further ensure that CK19-cre expression is limited to cholangiocytes/LPC, we exposed tamoxifen-injected *CK19-cre^+/−^*;p53*^Δ/Δ^*;Rb*^Δ/Δ^*;LSLR26R*^Δ/Δ^* mice to a 3,5-diethoxycarbonyl-1,4-dihydrocollidine (DDC) diet for three weeks to induce proliferation of cholangiocytes/LPC [30]. We observed LacZ expression only in cholangiocytes/LPC during DDC exposure and after switching back to a normal diet (Supplementary Figure S8A). These findings are in line with previously published findings which demonstrate that CK19-cre expressing cells are not able to generate hepatocytes even under stress conditions [31]

Importantly, RFA resulted in the formation of liver tumors at the site of tissue destruction in 42% of *CK19-cre^+/−^*;p53*^Δ/Δ^*;Rb*^Δ/Δ^* (Figure 7A; Table 1). Distinct neoplastic foci were detectable at first 9 weeks after RFA treatment and tumors showed strong LacZ expression and PCR analysis confirmed deletion of *p53* and *Rb* within these tumors (Figure 7A and Supplementary Figure S1A). Comparative histological analysis revealed that the tumors had the same morphology and expression pattern of epithelial and mesenchymal markers similar to tumors that developed in *Alb-cre^+/−^*;p53*^Δ/Δ^*;Rb*^Δ/Δ^* mice after PH or RFA (Figure 7B, and Supplementary Figure S8B). These findings provide strong evidence that the bile duct cell/LPC is the cell-of-origin of *p53^Δ/Δ^*;Rb*^Δ/Δ^* liver tumors induced by tissue destruction.

## DISCUSSION

An important question, which is difficult to address in human patients, is whether liver cancers originate from hepatocytes, liver progenitor cells or cholangiocytes. We used genetically modified mice that lack the expression of tumor suppressors in different liver cell lineages to identify the cell of origin in an injury-induced model of liver cancer. Albumin-cre activation in *p53/Rb* conditional knockout mice resulted in the expansion of structures with characteristics of bile ducts. We showed that *Albumin-cre* transgenic mice expresses cre not only in hepatocytes, but also in cholangiocytes/LPC. It is also possible that liver tumors originated from dedifferentiated hepatocytes in *Albumin-cre* transgenic mice, as it has been shown previously [32]. To genetically delete Rb and p53 exclusively in cholangiocytes/LPC we used the *Ck19-creERT* transgenic mice [29] and found that the same type of tumors developed at the same location. We and others have never observed any Ck19-cre expression in hepatocytes not even after DDC exposure (Supplementary Figure S8A, [31], indicating that CK19-cre expressing cells in this transgenic mouse line do not generate hepatocytes and also do not originate from dedifferentiated hepatocytes. These observations strongly suggest that p53/Rb deficient liver tumors in CK19-cre expressing mice originate from cholangiocytes/LPC at the injury site.

This study provides insights into the pathogenesis of the development of liver tumors after surgical resection or RFA treatment, a scenario that is often observed in human liver cancer patients that underwent surgical or RFA therapy [6]. We provide now strong evidence that the necrotic injury site induced by these therapeutic modalities could serves as an optimal microenvironment for the initiation of liver tumors. Notably, surgical resection and RFA caused liver cancer only in p53 and Rb deficient livers, while wild type mice that underwent similar procedure were tumor-free. Nevertheless, p53 and Rb are commonly inactivated in human liver cells for example through infection with hepatitis B and C virus [33–35], raising the risk to develop liver tumors after surgical interventions. In fact, a recent study showed that liver cancer patients (n=266) infected with Hepatitis B virus had a 2-year recurrence rate of 54.3% after RFA [36]. Furthermore a study with 72 HCC patients revealed that a high hepatitis B virus load (>2000IU/ml) at the time of tumor resection was the most important risk factor for HCC recurrence after 19 months (p=0.001) [34]. Moreover antiviral treatment after resection was associated with significantly lower cumulative risk of recurrence [36, 37].

We show that thermal destruction leads to massive coagulation necrosis, followed by a wound healing response characterized by the migration of activated cholangiocytes/LPC, inflammatory cells, myofibroblasts, and deposition of extracellular matrix (fibrosis) into the necrotic regions. In wild type livers this dynamic wound healing process slows down and results in sequestration of the necrotic regions. In contrast, the chronic inflammatory response continues in p53/Rb deficient livers and leads to further expansion and enhanced proliferation of cholangiocytes. Remarkably, within 5 weeks after injury, cholangiocytes/LPC transform and initiate tumor formation within the necrotic regions. These migrating and expanding bile ducts were surrounded by cells that expressed high levels of TGFβ. Based on the morphology and the presence of brown pigment in the cytoplasm, the TGFβ-expressing cells represent most likely macrophages that were possibly recruited to injury site to phagocytize the necrotic debris (Figure 5A). Previous studies have shown that an increased TGFβ gradient promotes differentiation towards biliary lineage [16, 17, 25, 38] which is consistent with our finding that we only observed migration and expansion of cholangiocytes, and never observed any hepatocytes in the necrotic region. When we exposed isolated cholangiocytes/LPC to different dosages of TGFβ, we observed that Rb/p53 deficient cells proliferate more efficiently than wild type cells. These findings are also in line with the observation that the TGFβ-receptor contributes to the development of liver tumors in *p53* deficient livers [16]. In addition, TGFβ is well known for inducing EMT in cancer cells [39], a phenotype that we clearly observed in the tumors that developed at necrotic injury where many cells express high levels of TGFβ. Together, our studies provide strong evidence for a model in which the increased TGF-β gradient generated by macrophages at the necrotic injury site promotes the migration, expansion, transformation and EMT of *p53* and *Rb* deficient cholangiocytes/LPC.

Surprisingly, the use of anti-inflammatory agents showed that the overall tumor incidence after RFA was not significantly reduced, suggesting that other mechanism such as hypoxia may contribute to peri-necrotic tumor formation. Our *in vitro* study with CK19+ isolated cells (Figure 5E and Supplementary Figure S9A, S9B) as well as our previous studies utilizing an *in vivo* colon carcinoma cancer model [40] provide strong evidence that hypoxia could contribute to the positive selection cancer cells at the injury site.

In summary, our findings show that deletion of *p53* or *Rb* leads to the expansion, transformation, and EMT of bile duct cells/LPC within an inflammatory, hypoxic and necrotic environment induced by surgery/RFA. We conclude that the combination of loss of tumor suppressors, creation of hypoxia/necrosis and the accompanied activation of TGFβ signaling causes cancer and EMT at the surgery site.

Finally, we provide the field with a novel mouse model, in which surgical resection and RFA treatment leads to formation of highly aggressive, fast growing and undifferentiated carcinomas. This model could represent a tool to test the efficacy of anti-cancer drugs that are aimed to use in human patients that developed liver tumors after hepatic resection or RFA.

## MATERIALS AND METHODS

### Animals and histology

All experiments were approved by the Utrecht University Animal Ethics Committee and performed according to institutional and national guidelines. *Albumin-cre* and *R26R-LacZ^f/f^* mice were derived from Jackson laboratory [22, 23]. *Rb^f/f^* and *p53^f/f^* mice were provided by Dr. A. Berns (Netherlands Cancer Institute, Amsterdam, The Netherlands). *Ck19-cre-ERT* mice were generated as described previously [22]. Partial hepatectomy (PH) and RFA experiments were performed on mice aged for 10-14 weeks. First PH was done as described previously [41]. The second PH, removing the right lateral lobe, was performed 10 weeks after the first PH [42]. RFA was performed as described previously [40]. X-gal and immunostaining on liver sections were performed as described previously [43].

Additional description of materials and methods is provided as supplementary information.

## SUPPLEMENTARY MATERIALS AND METHODS


