# Supplementary material for: Surgical resection and radiofrequency ablation initiate cancer in cytokeratin-19+- liver cells deficient for p53 and Rb
Source: Oncotarget. 2016 Jun 13;7(34):54662–75. doi: 10.18632/oncotarget.9952 (PMC5342371; doi:10.18632/oncotarget.9952)
Supplement: Supplementary file 1 [file oncotarget-07-54662-s001.pdf]

Surgical resection and radiofrequency ablation initiate cancer in cytokeratin-19+- liver cells deficient for p53 and Rb

SUPPLEMENTARY MATERIALS AND METHODS

Animals

The induction of CreERT expression was induced by intraperitoneal injection of Tamoxifen (Sigma, T5648-1G, 15mg in corn oil per mouse divided in five serial injections). Mice were injected at the age of 10 weeks shortly before RFA (Day-5 , Day -3), during RFA (Day 0), and after RFA (Day +2, Day +4). To induce oval cell proliferation, 10 week old mice were given a 3,5-diethoxycarbonyl-1,4-dihydrocollidine (0.1% DDC, Bio-Serv) diet for three weeks. The induction of CreERT expression in DDC exposed mice was induced by four tamoxifen injections at the age of 3, 10, 11, 12 weeks (13 mg total).

Liver organoids

Organoids isolated from bile duct fragments of wild type (WT) and p53/Rb deficient liver were cultured as described before [1]. To investigate proliferation of WT vs p53/Rb deficient liver organoids, 5-ethynyl-2′-deoxyuridine (Edu) (Invitrogen, A10044) was incorporated for 2 hours at 37°C from day 2 until day 5 of culture. To investigate the effect of TGF-β on liver organoid proliferation, organoids were incubated for 24 hours with TGF-β (R&D systems, 7666-MB-005) at concentration of 0, 5 and 10 ng/ml respectively. Then organoids were pulsed with Edu for 2 hours. For hypoxia experiments, organoids were incubated for 24 hours in humidified chamber at 37°C, 5% CO_2 and 2.5% O_2 in Don Whitley H35 hypoxystation (Don Whitley Scientific, UK). Organoids cultured under normoxia were maintained in a standard cell culture incubator. For oxidative stress experiments, organoids were incubated with or without 1.25 µM *N*-acetylcysteine (Sigma Aldrich) and 1mM Hydrogen peroxide (Merck Millipore) for 24 hours .

EDU-incorporation assays and trypan blue analysis

After 18hours, organoids cultured under normoxia, hypoxia and oxidative stress experiments were incubated with EdU for 6hours, and then organoids were analyzed for trypan blue exclusion using TC20 automated cell counter (Bio-rad). For EdU analysis, cells were fixed with 4% paraformaldehyde and processed in a click reaction with 5 µM Alexa Fluor 488 azide (Life Technologies, A10266) according to manufacturer’s instruction. Nuclei were stained with DAPI (Sigma-Aldrich, D9542). The percentage of EdU positive cells was analyzed using automated image acquisition and data analysis with the Cellomics ArrayScan VTI HCS Reader (Thermo Scientific) and confocal SP II microscope (Leica Microsystems)

Flow cytometry analysis

Organoids were trypsinized and fixed with ice cold 70% ethanol in PBS and stored in the fridge. Prior to FACS analysis, nuclei were stained with Propidium iodide as described previously [2] and analyzed with FACS Calibur and CellQuest software (BD Bioscience). Histograms overlay were created using FlowJo software.

β-galactosidase and immunostaining

For antigen retrieval we used either the citrate buffer at pH 6 (S100A4, Ki67, CK19) or the Tris-EDTA buffer at pH 9 (HNF4α , E-cadherin). The following antibodies were used: rabbit anti CK-19 (generated by Wouter Lamers, Academic Medical Center, Amsterdam, The Netherlands, 1:1500 in PBS); the hybridoma rat anti-CK19 (Troma III) developed by Rolf Kemler was obtained from the Developmental Studies Hybridoma Bank developed under the auspices of the NICHD and maintained by The University of Iowa, 1:20 in PBS; rabbit anti-Ki67 (Labvision/Neomarkers, RM-9106-S0, 1:75 in PBS); rabbit anti-HNF4α (Santa Cruz, Sc-8987, 1:200 in 10% goat serum); rabbit anti-S100A4 (Dako, A5114, 1:200 in PBS); goat anti-vimentin (Santa Cruz, Sc-7557, 1:50 in PBS/Tween); mouse anti-E-cadherin (BD Bioscience, 610182, 1:500 in MOM diluent); mouse on mouse (MOM) basic kit (Vector Laboratories, BMK-2202). In all immunohistochemical staining procedure except for E-Cadherin, biotinylated goat anti rabbit (Vector Laboratories, BA-1000,1:250 in PBS or 5% goat serum for HNF4a) was used. Fluorescence labeled goat anti-rat IgG Alexa Fluor 488 (Invitrogen, A11006), goat anti-rabbit IgG Alexa Fluor 568 (Invitrogen, A11036) and donkey anti-goat IgG alexafluor 568 (Invitrogen, A11057) were diluted in 5% mouse serum in PBS containing 0.05% Tween to make a final dilution of 1:100. 5-Bromo-4-chloro-3-indolyl β-D-galactopyranoside (Sigma-Aldrich, B4252-100MG, 1mg/ml in PBS) was used for enzymatic detection of β-galactosidase.

Histological image acquisition and processing

Haematoxylin and eosin as well as immunohistochemistry images were acquired using DP25 camera, Labsens soft imaging version 1.1 and Olympus BX45 microscope (Olympus, Zoeterwoude, The Netherlands). Immunofluorescence images were acquired using Leica DFC 425c camera and Leica Application Suite version 4 (Leica Microsystems B.V. Rijswijk, The Netherlands, and BX60 microscope (Olympus, Zoeterwoude, The Netherlands). Images were processed using Adobe photoshop cs6/CC and exported to Adobe illustrator cs6/CC (Adobe Systems, Inc., San Jose, California).

Pathological analysis of liver tissues

Pathological analysis was performed by a board-certified veterinary pathologist (A.d.B.) and a human pathologist (T.R.). Liver tumors were classified according to the nomenclature and diagnostic criteria for hepatobiliary lesions in rats and mice [3].

Genotype analysis

Genotyping on livers and liver tumors was performed using allele-specific primers. The following primers have been used: *p53*F: AAGGGGTATGAGGGACAAGG, *p53*R:GAAGACAGAAAAGGGGAGGG, *p53*F1(int):CACAAAAACAGGTTAAACCCA, *Rb*F: CTCAAGAGCTCAGACTCATGG, *Rb*R:GGCGTGTGCCATCAATG, *Rb*212: GAAAGGAAAGTCAGGGACATTGGG; *Cre*F:ATGCTTCTGTCCGTTTGCCG, *Cre*R:CCTGTTTTGCACGTTCACCG; *Ck19-cre*Pr2: GTTCTTGCGAACC TCATCACTC, *Ck19-cre* Pr3: GCAGAATCGCCAGGAATTGACC.

Statistics

Survival curve was drawn using SPSS statistical package and statistical difference was calculated using SPSS log rank test. Statistical test used in table is student's t-test; statistical test for bar graphs was computed using Mann-Whitney U test, mean, standard deviation and standard error of the mean were calculated using Microsoft excel (2010) ®.

REFERENCES

1. Huch M, Dorrell C, Boj SF, van Es JH, Li VS, van de Wetering M, Sato T, Hamer K, Sasaki N, Finegold MJ. In vitro expansion of single Lgr5^{+} liver stem cells induced by Wnt-driven regeneration. Nature. 2013; 494: 247-250.
2. Pandit SK, Westendorp B, Nantasanti S, van Liere E, Tooten PC, Cornelissen PW, Toussaint MJ, Lamers WH, de Bruin A. E2F8 is essential for polyploidization in mammalian cells. Nature cell biology. 2012; 14: 1181-1191.
3. Thoolen B, Maronpot RR, Harada T, Nyska A, Rousseaux C, Nolte T, Malarkey DE, Kaufmann W, Kuttler K, Deschl U, Nakae D, Gregson R, Vinlove MP, et al. Proliferative and nonproliferative lesions of the rat and mouse hepatobiliary system. Toxicologic pathology. 2010; 38: 5S-81S.

SUPPLEMENTARY FIGURES

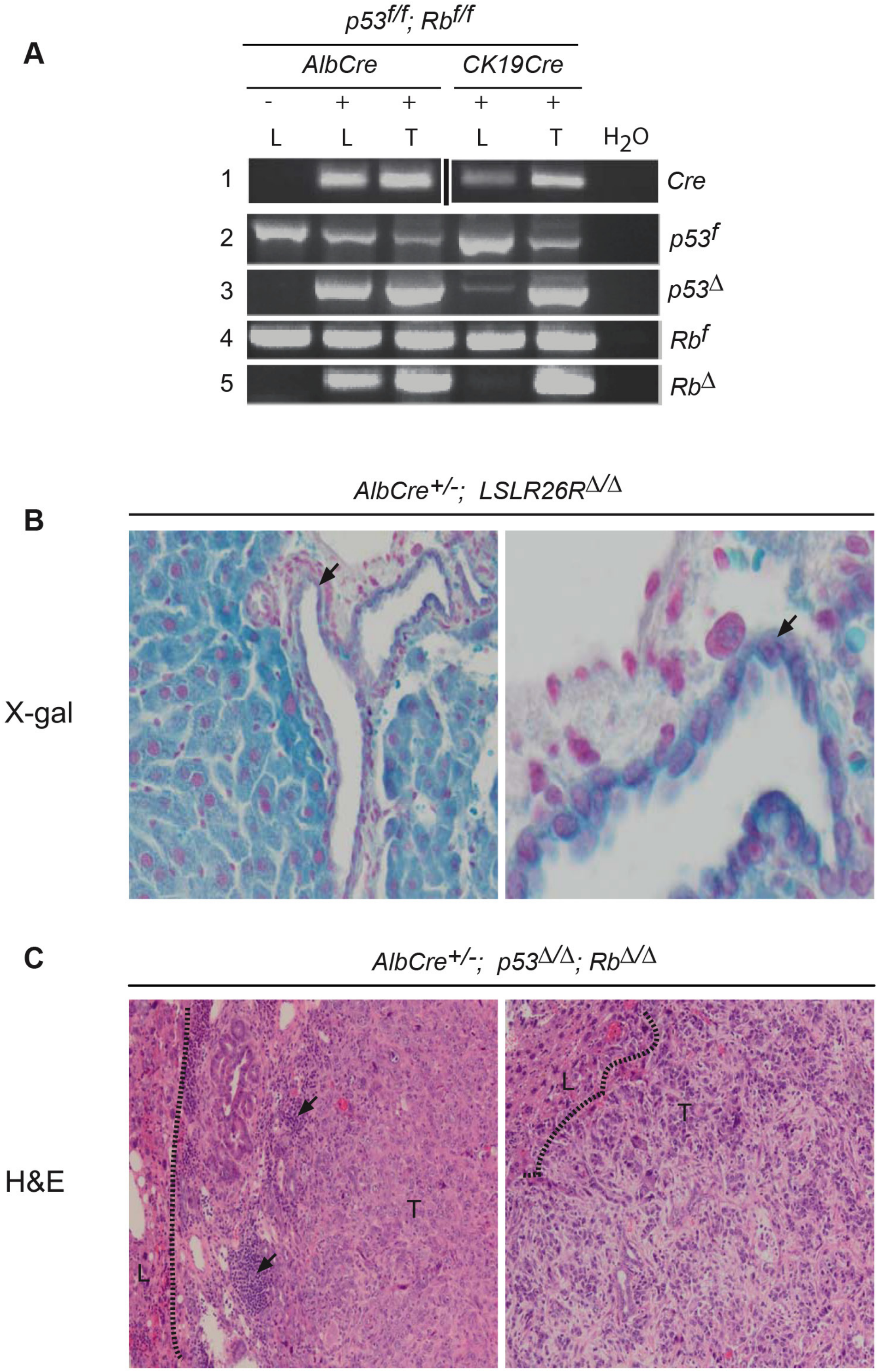

Supplementary Figure S1: A. Genotyping PCR on livers (L) and tumors (T). $^{\Delta}$ stands for deleted, and^{f} for floxed alleles. Lane 1, left and right images taken from separate gels. **B.** LacZ reporter in normal liver left and right picture at 100x and 400x magnifications respectively. **C.** H&E images from undifferentiated liver tumor. Arrowheads indicate infiltrating inflammatory cells. Images at 100x magnification.

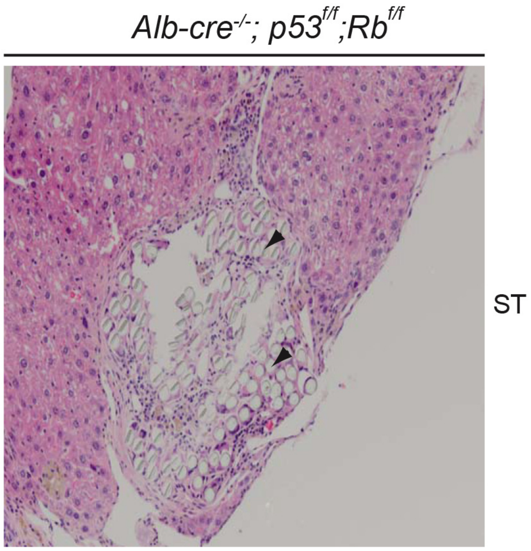

Supplementary Figure S2: Representative picture of H&E stained liver section from a stump (ST) of a control ***Alb-cre$^{-/-}$; p53$^{f/f}$; Rb$^{f/f}$*** **mouse that underwent partial hepatectomy.** Arrowheads indicate location of suture material. 100x magnification.

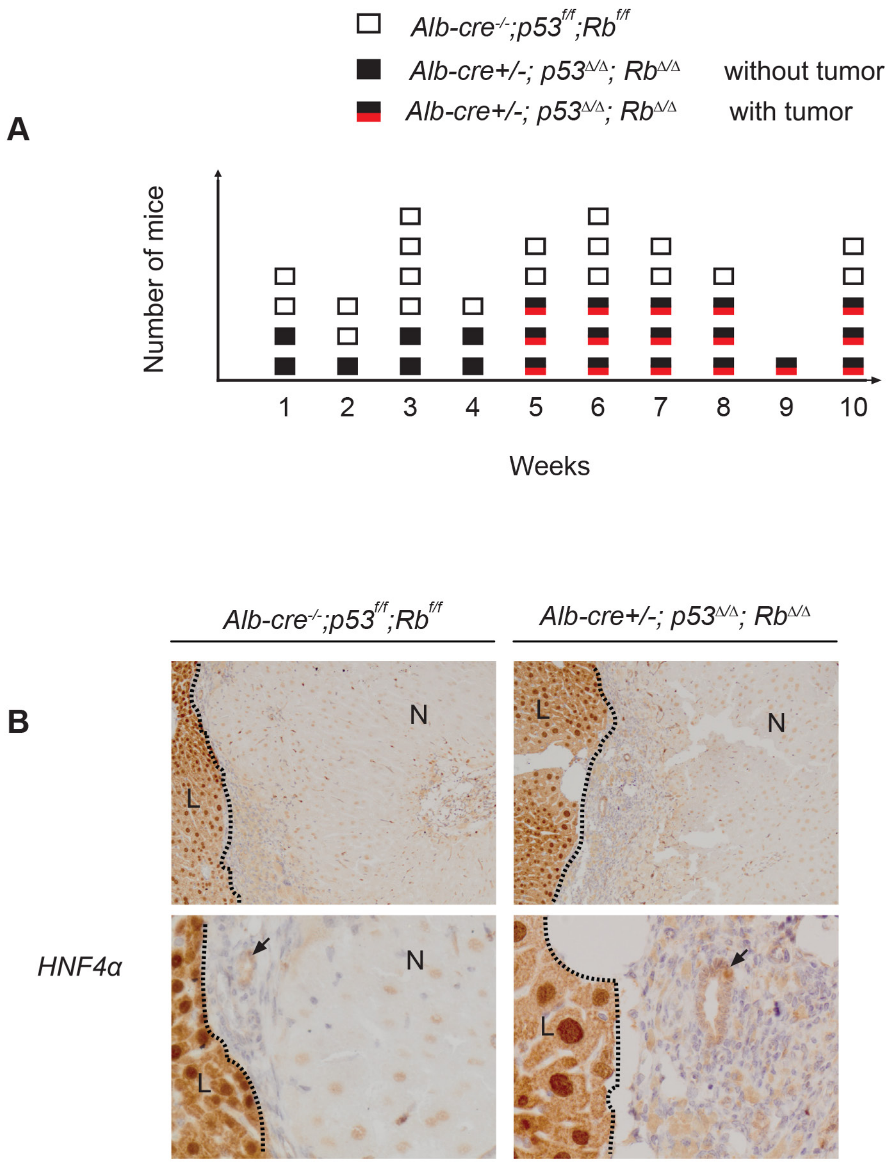

Supplementary Figure S3: A. Tumor incidence, **B.** HNF4α analysis, 3 weeks after RFA. Viable liver (L), necrotic (N) RFA lesion. Bile ducts are indicated by arrows. Magnifications in [(A), (B)]: upper panels 100x, lower panel 400x.

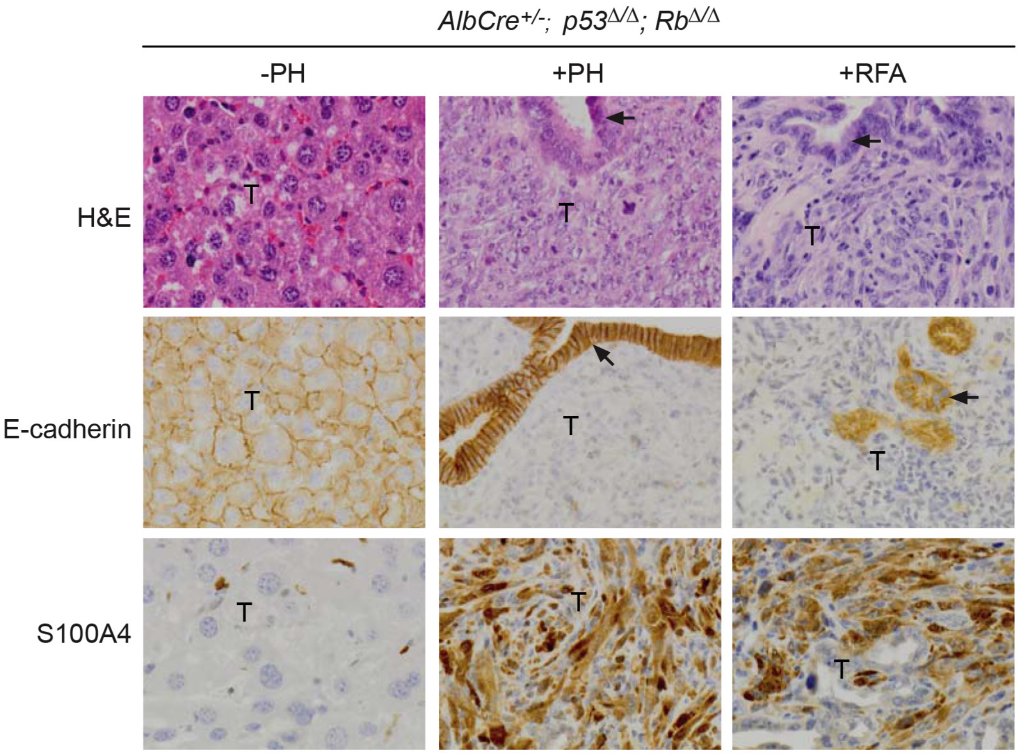

Supplementary Figure S4: Higher magnification (400x) of the epithelial and mesenchymal marker analysis on undifferentiated carcinomas in *Alb-cre*$^{+/-}$*; p53*$^{\Delta/\Delta}$*; Rb*$^{\Delta/\Delta}$ **mice.** Representative pictures of H&E and immunohistochemical staining for E-cadherin, and S100A4.

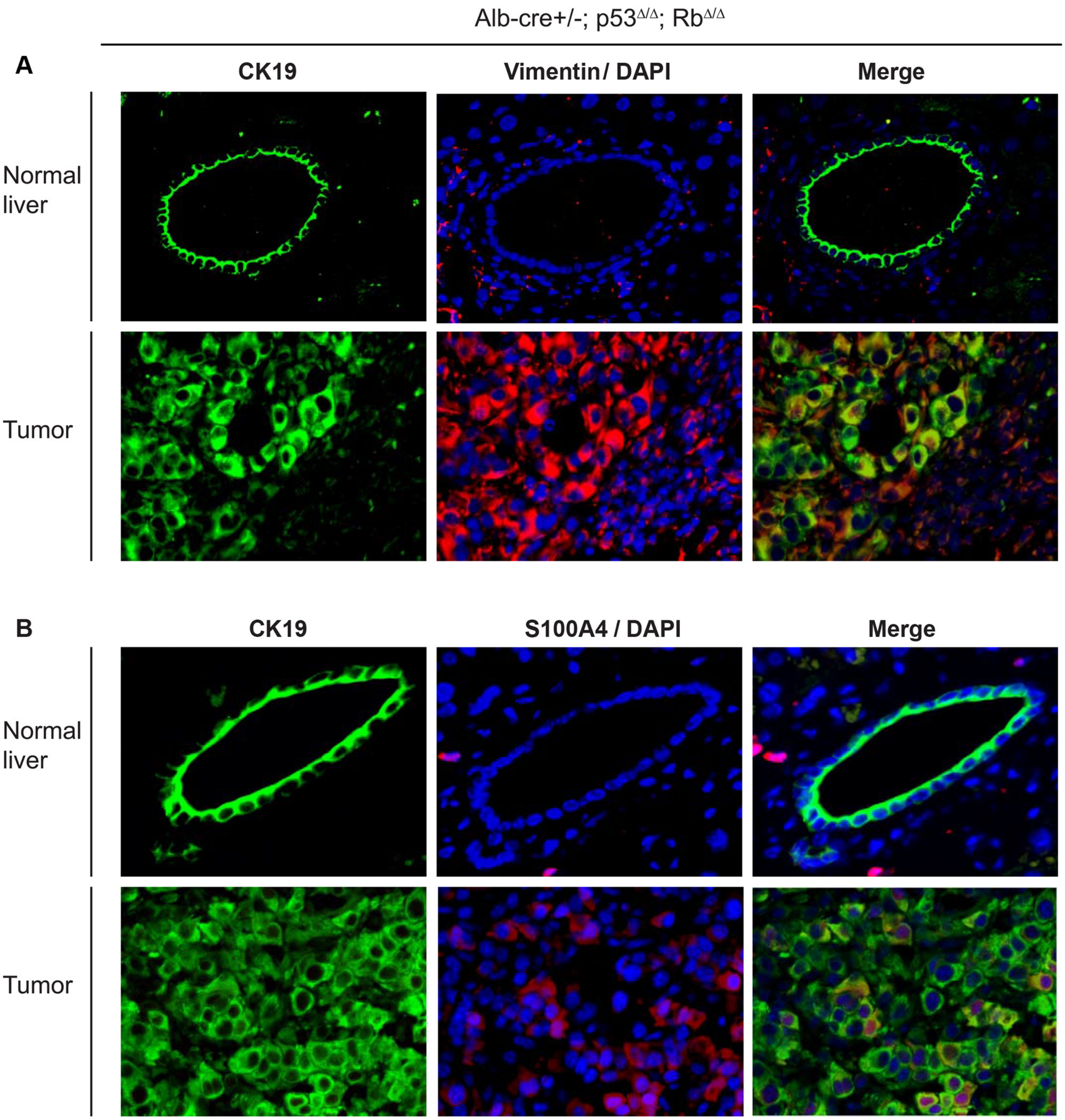

Supplementary Figure S5: Immunofluorescence images (400x magnification). A. Normal liver; top row and tumors at the bottom row showing epithelial marker CK19 (green), mesenchymal marker vimentin (red), and merged CK19 and vimentin (yellow). **B.** Normal liver; top and tumors at the bottom row showing epithelial marker CK19 (green), mesenchymal marker S100A4 (red), and merged CK19 and S100A4 (yellow).

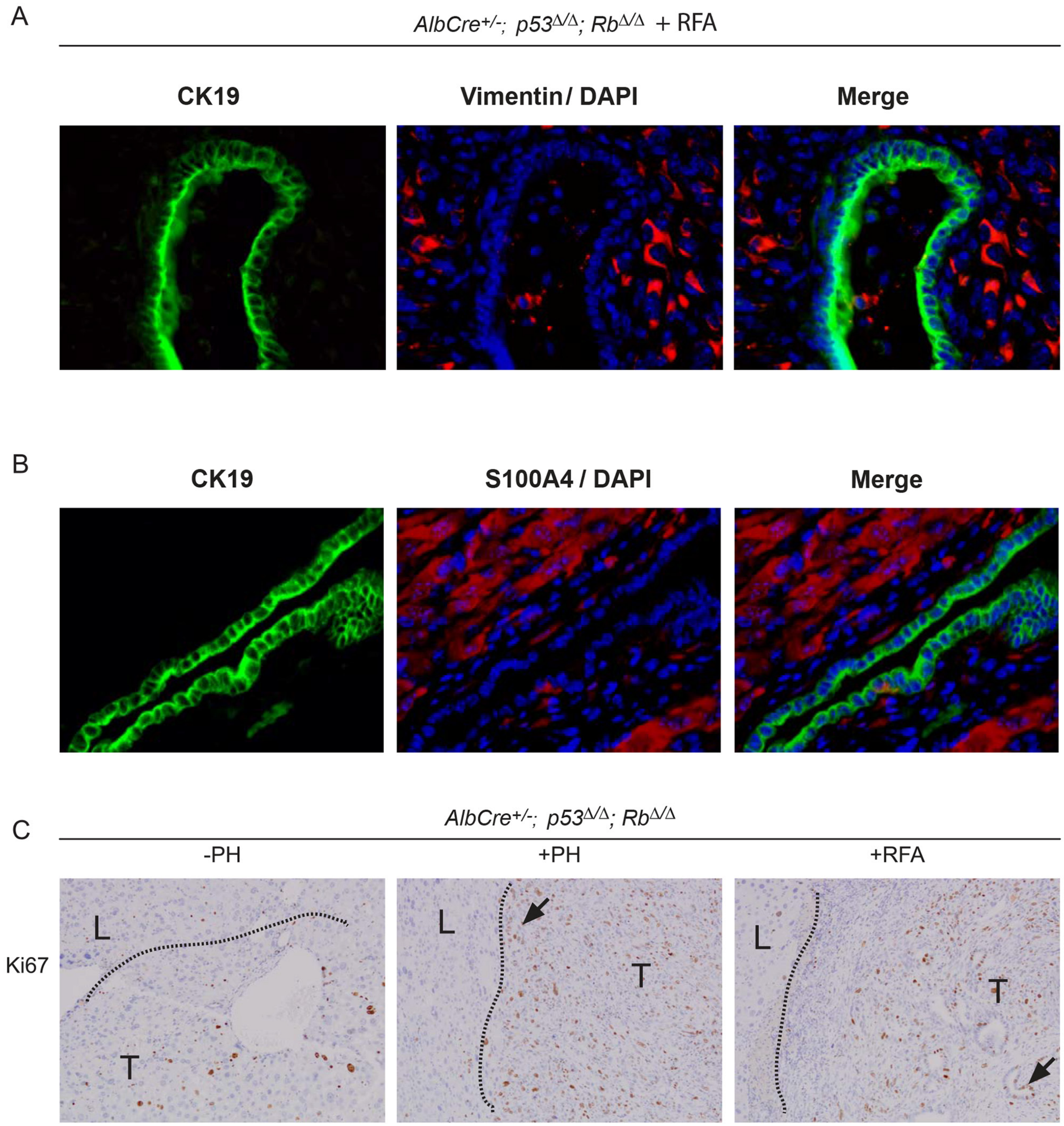

Supplementary Figure S6: A, B. Immunofluorescence images of well differentiated bile ducts positive for CK19 and surrounded by CK19 negative mesenchymal tissues which are positive for mesenchymal markers vimentin and S100A4 respectively. **C.** Ki67 on liver (L), images (100x magnification) from well differentiated tumor **T.** without surgery and undifferentiated carcinomas from liver with partial hepatectomy (+PH) or RFA. Ki67 positive bile ducts are shown (arrow).

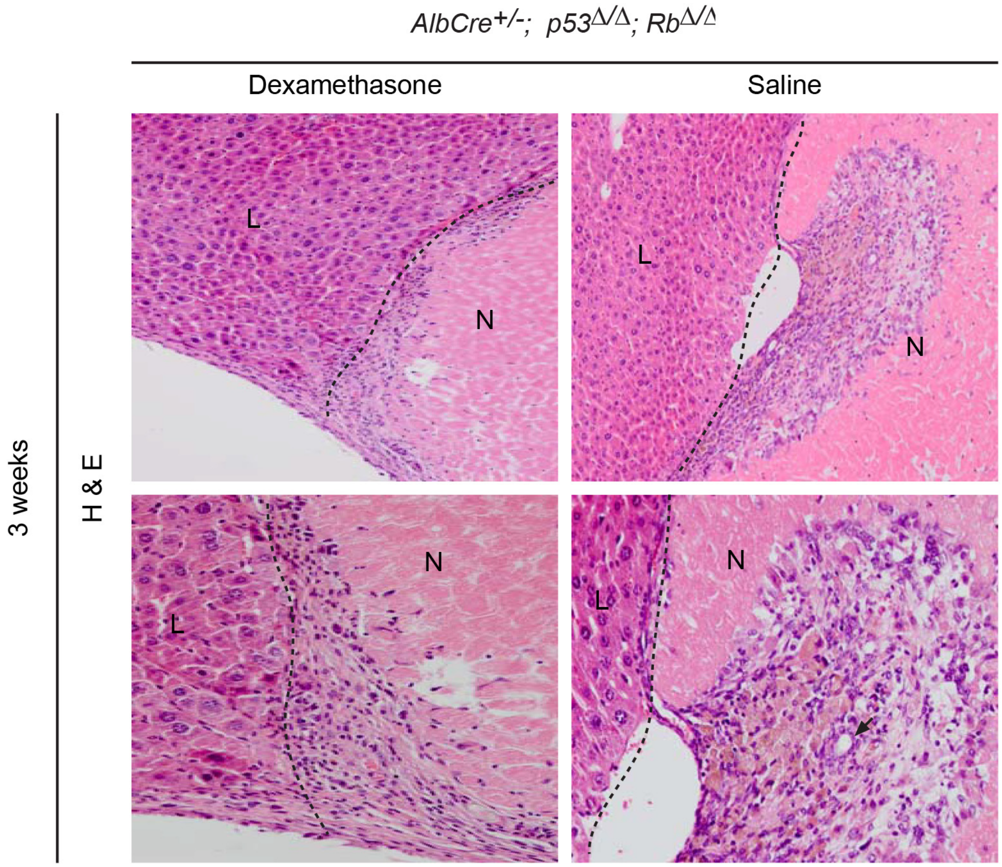

Supplementary Figure S7: H &E staining of liver tissues from mice treated with dexamethasone (left column) and control mice received saline treatment (right column). Mice received saline treatment show increased inflammatory reaction and migration of bile ducts into the necrotic region 3 weeks after RFA. Arrow indicate bile duct in the necrotic region, images magnification at 100x (top row) and 200x (bottom row) respectively.

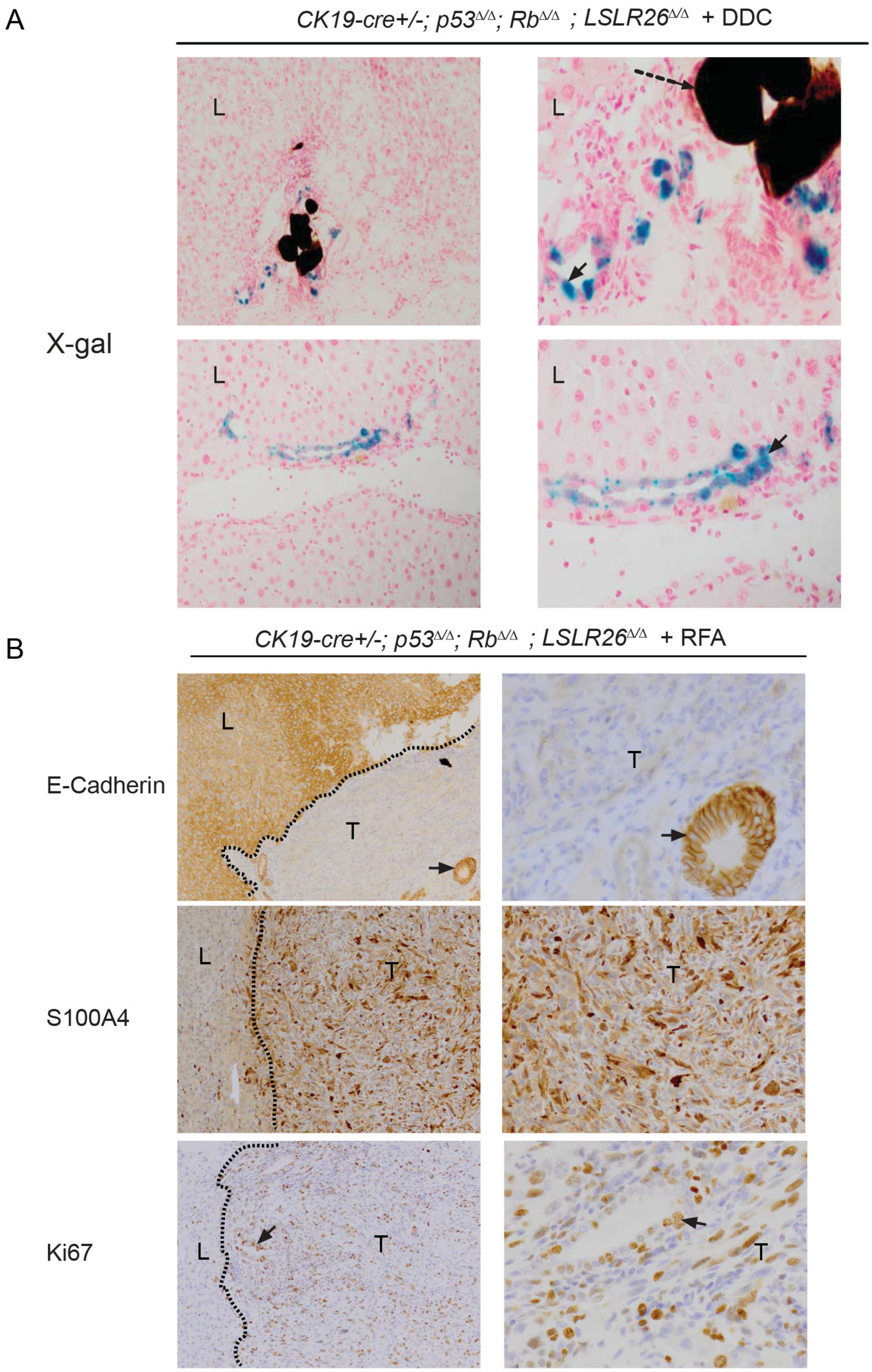

Supplementary Figure S8: A. X-gal staining of liver of *CK19-Cre*$^{+/-}$*; p53*$^{\Delta/\Delta}$*; Rb*$^{\Delta/\Delta}$ *; LSLR26*$^{\Delta/\Delta}$ mice treated with DDC for 3 weeks (active phase) (top panel), arrow indicate porphyrin in portal region. Bottom panel is liver from mice fed with DDC for 3 weeks (active phase) and then switched to normal diet for 10 weeks (recovery phase). In both active and recovery phases, only bile ducts are positive for X-gal and not activated liver progenitor cells or surrounding mesenchymal tissues. **B.** Undifferentiated tumor cells show loss of epithelial marker E-cadherin and gain of mesenchymal marker S100A4. Bile duct cells (arrow) and surrounding tumor cells show high levels of Ki67 staining. Images magnifications: 100x (left panel), and 400x (right panel).

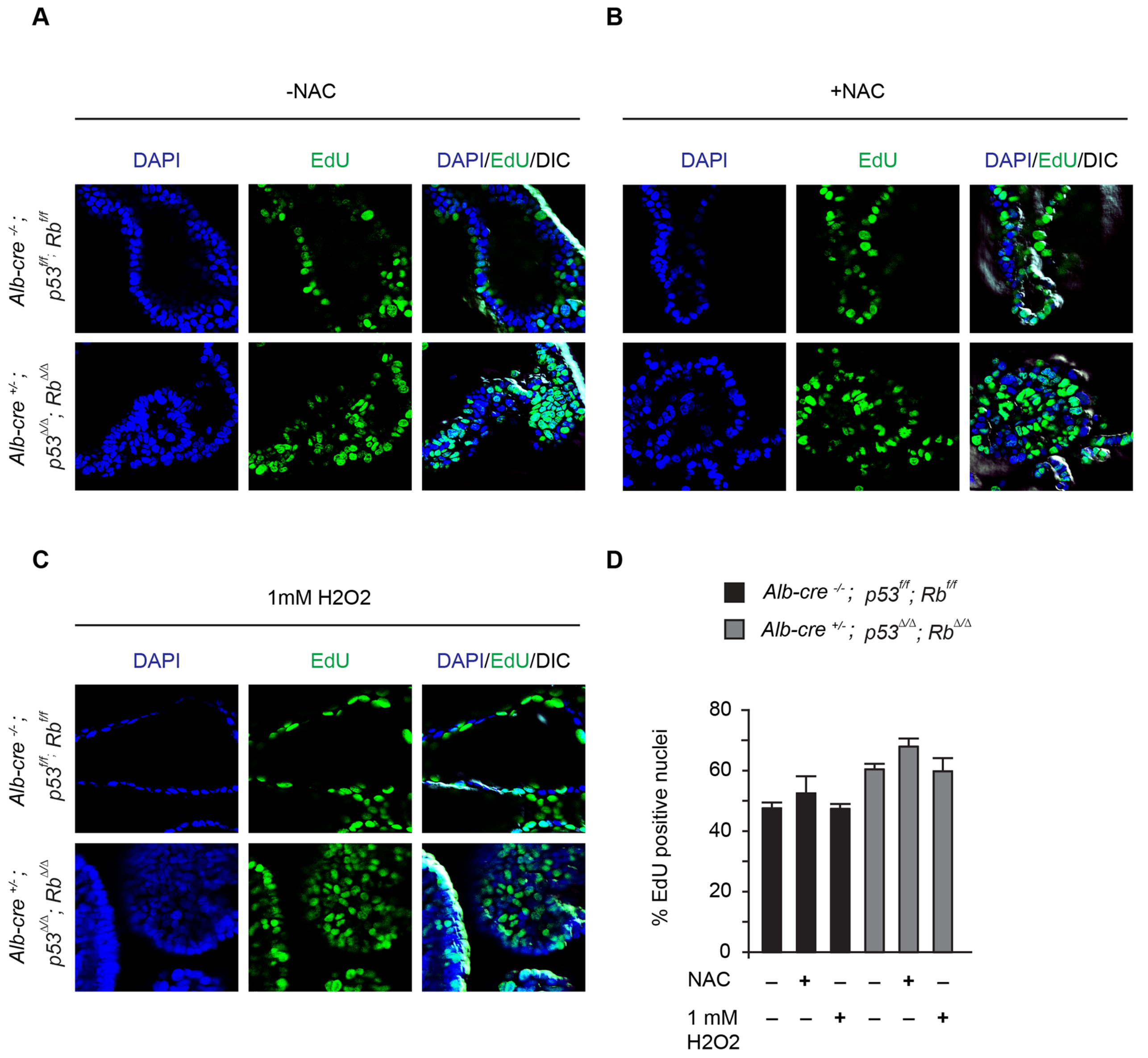

Supplementary Figure S9: A. *Alb-cre*$^{-/-}$*;p53*$^{f/f}$*;Rb*$^{f/f}$ and *Alb-cre*$^{+/-}$*; p53*$^{\Delta/\Delta}$*; Rb*$^{\Delta/\Delta}$ organoids cultured in absence of *N*-acetylcysteine (NAC). **B.** *Alb-cre*$^{-/-}$*;p53*$^{f/f}$*;Rb*$^{f/f}$ and *Alb-cre*$^{+/-}$*; p53*$^{\Delta/\Delta}$*; Rb*$^{\Delta/\Delta}$ organoids cultured in presence of 1.25 μ M NAC. **C.** *Alb-cre*$^{-/-}$*;p53*$^{f/f}$*;Rb*$^{f/f}$ and *Alb-cre*$^{+/-}$*; p53*$^{\Delta/\Delta}$*; Rb*$^{\Delta/\Delta}$ organoids treated with 1 mM H2O2 in absence of NAC. **D.** Quantification of EdU positive nuclei, histograms represents mean + standard deviation, *$p < 0.05$.

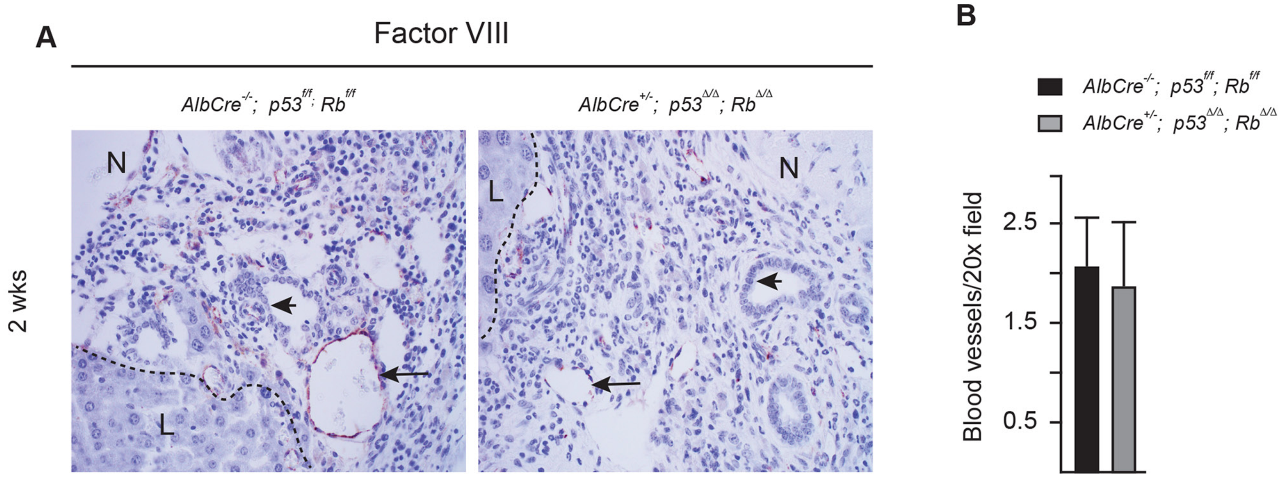

Supplementary Figure S10: A. Bile ducts (arrow head) and blood vessels lined by endothelial cells stained with Factor VIII (arrow). **B.** Number of blood vessels per filed at 20x objective lens in transition zone between liver (L) and necrotic RFA region (N) in *Alb-cre*$^{-/-}$*;p53*$^{f/f}$*;Rb*$^{f/f}$ and *Alb-cre*$^{+/-}$*; p53*$^{\Delta/\Delta}$*; Rb*$^{\Delta/\Delta}$ liver, 2 weeks after RFA. Histograms represents mean + standard deviation.

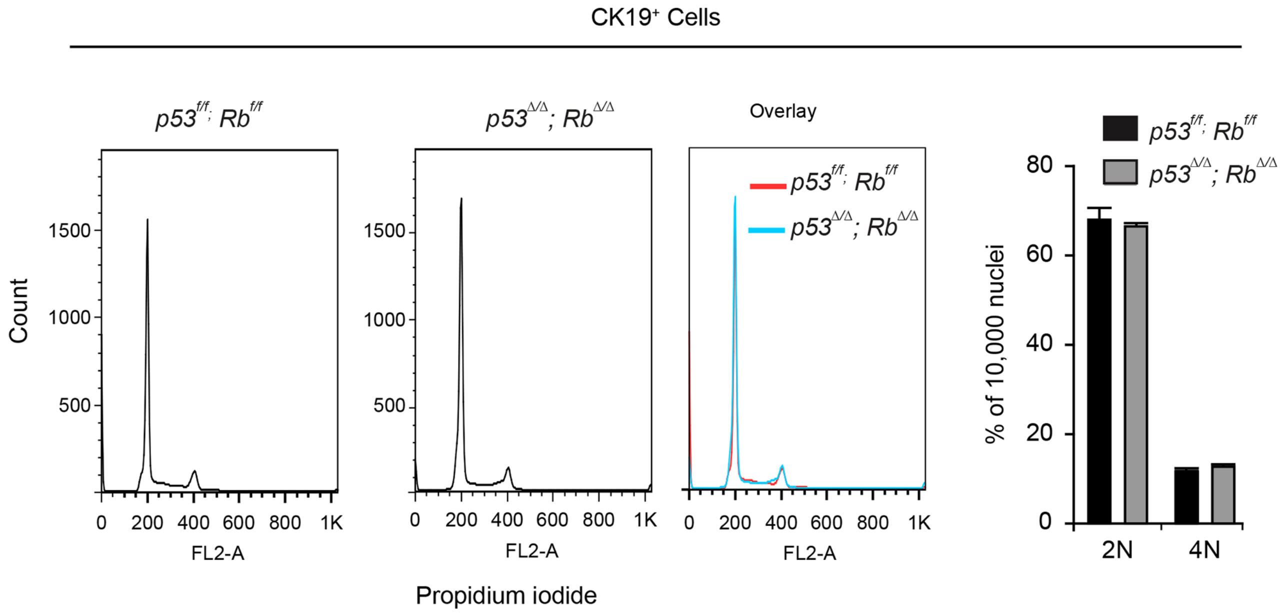

Supplementary Figure S11: Histograms of flow cytometry data of *Alb-cre*$^{-/-}$*;p53*$^{f/f}$*;Rb*$^{f/f}$ **(***p53*$^{f/f}$*;Rb*$^{f/f}$ **) and** *Alb-cre*$^{+/-}$*; p53*$^{\Delta/\Delta}$*; Rb*$^{\Delta/\Delta}$ **(***p53*$^{\Delta/\Delta}$*; Rb*$^{\Delta/\Delta}$ **) of CK19+ organoids representing counts of diploid and tetraploid nuclei per 10,000 gated nuclei, and % of diploid and tetraploid nuclei, Black and grey bar histograms represents mean + standard deviation.**
